# Myeloid-Derived Suppressor Cells in Sepsis

**DOI:** 10.1155/2014/598654

**Published:** 2014-06-03

**Authors:** Dengming Lai, Chaojin Qin, Qiang Shu

**Affiliations:** ^1^Department of Thoracic & Cardiovascular Surgery, Children's Hospital, Medical College, Zhejiang University, Hangzhou 310003, China; ^2^Key Laboratory of Reproductive Genetics (Zhejiang University), Ministry of Education, Hangzhou 310003, China

## Abstract

Sepsis is a systemic, deleterious host response to widespread infection. Patients with sepsis will have documented or suspected infection which can progress to a state of septic shock or acute organ dysfunction. Since sepsis is responsible for nearly 3 million cases per year in China and severe sepsis is a common, expensive fatal condition in America, developing new therapies becomes a significant and worthwhile challenge. Clinical research has shown that sepsis-associated immunosuppression plays a central role in patient mortality, and targeted immune-enhancing therapy may be an effective treatment approach in these patients. As part of the inflammatory response during sepsis, there are elevations in the number of myeloid-derived suppressor cells (MDSCs). MDSCs are a heterogeneous population of immature myeloid cells that possess immunosuppressive activities via suppressing T-cell proliferation and activation. The role of MDSCs in sepsis remains uncertain. Some believe activated MDSCs are beneficial to the sepsis host by increasing innate immune responses and antimicrobial activities, while others think expansion of MDSCs leads to adaptive immune suppression and secondary infection. Herein, we discuss the complex role of MDSCs in immune regulation during sepsis, as well as the potential to target these cells for therapeutic benefit.

## 1. Introduction

Over the last decade, during the emergence of the “host theory,” it was first assumed that the clinical features of sepsis were the result of an uncontrolled inflammatory response and an increase in inflammatory cytokines, commonly referred to as a “cytokine storm” [1]. Patients with sepsis may present with hypothermia, shock, elevated heart rate, altered mental status, tachycardia, an elevated white-cell count, and acute organ dysfunction [2, 3]. Recently, researchers have advanced this theory and suggested that infection triggers a much more complex, variable, and prolonged host response in which both proinflammatory and anti-inflammatory cytokines grow rapidly in number. Additionally, some patients with sepsis rapidly produce both categories of cytokines, whereas others have either a predominance of anti-inflammatory cytokines or globally depressed cytokine production [4]. Many investigative agents have been examined in an effort to downregulate cytokine release, which led to the survival of the majority of patients in the early period of this syndrome. Unfortunately, the patients who survive are at risk for developing nosocomial infections with organisms not typically pathogenic in immunocompetent hosts and can have reactivation of latent viruses [5, 6]. The failure of several clinical trials in sepsis has led researchers to be firmly convinced that future research needs to take a new direction [7–9]. Experts have delineated reasons for the failures of new investigative drugs and have presented some advice in the design and conduct of sepsis trials. For example, previous clinical drug studies tested drugs in young and healthy animal models, but patients frequently present with coexisting illness. The outcomes in patients are poorer than those in animals, so animal models of aging and preexisting disease are needed. Torgersen and colleagues have highlighted key immunological defects that impair host immunity including impairment of splenocyte function and depletion of immune effector cells [10]. Additionally, their clinical studies have found that ICU patients whose deaths are sepsis-related have biochemical, flow cytometric, and immunohistochemical findings consistent with immunosuppression [11]. Therefore, we must pay close attention to the immunosuppression component of sepsis.

MDSCs possess the ability to suppress antigen-specific CD8^+^ and CD4^+^ T-cell activation, and several studies have found that MDSCs dramatically increase with sepsis [12]. The role of MDSCs in sepsis is not fully understood. It has been proposed that the overall role of MDSCs involves much more than simply being an immunosuppressive population. Rather, MDSCs expansion is a common response in all inflammatory processes and the expansion of MDSCs population may be protective for the host by increasing immune surveillance and innate immune responses [13]. Exploring the relationship between sepsis and MDSCs will provide insight into how these cells function and guide the development of future treatments for sepsis in clinical trials.

## 2. Sepsis Etiology: A Central Role of Host Immunity

Sepsis is one of the oldest and most elusive syndromes in medical history. In China, the number of patients with sepsis may exceed 3,000,000 per year with the true incidence being presumably higher, and our group reported an 8.68% occurrence rate of severe sepsis in surgical ICUs in China, with a hospital mortality rate of 48.7% [14]. Studies from the United States show a similar rate of sepsis in the general population [15]. Although many treatments have been applied and many of the treatment successes are owed to antibiotic therapy, the mortality of sepsis still remains at about 20% to 30%. High mortality rates have drawn attention to the incidence of sepsis within populations. The incidence of sepsis depends on both the characteristics of the invasive pathogen and the state of the host immune system. Epidemiologic studies have shown that pneumonia is the most common etiology, followed by intra-abdominal and urinary tract infections [15, 16]. Typically, blood cultures are positive in only one third of cases [15, 17, 18].* Staphylococcus aureus* and* Streptococcus pneumonia* are the two most common gram-positive isolates, whereas* Escherichia coli*,* Klebsiella* species, and* Pseudomonas aeruginosa* predominate among gram-negative isolates, with a greater total incidence of gram-positive infections [17, 19]. In a recent study, gram-negative bacteria were isolated in 62% of patients who had positive cultures, 47% with gram-positive bacteria and 19% with fungi [20].

When pathogens invade the host, they will activate immune cells through an interaction with pattern-recognition receptors. These receptors recognize structures that are conserved among microbial species, which leads to the upregulation of inflammatory gene transcription and the initiation of innate immunity. Phagocytes like monocytes, macrophages, neutrophil granulocytes, and MDSCs can migrate to the infectious tissues and secrete anti-inflammatory cytokines. They phagocytize the pathogens, promote tissue repair, induce regulatory T-cells, and reduce inflammation. When sepsis initiates, the abilities of immune effector cells are strongly impaired, along with antigen-specific primary antibody production [21]. Some patients may become infected with a pathogen due to a weakened immune system. Researchers have shown that patients who survive early sepsis, but remain dependent on intensive care, have immunosuppression, which is evidenced by a reduced expression of HLA-DR on myeloid cells [11]. These patients have been found to have ongoing infectious foci or reactivation of latent viral infections, despite antimicrobial therapy [6, 10]. Other scholars have found that patients who died of sepsis in the ICU encountered strong functional impairments of splenocytes and the lungs also had increased expression of MDSCs phenotypes from sepsis versus control tissue (47.9% versus 15.7%) [11].

## 3. The Derivation and Subsets of MDSCs

In the early 1900s, these cells gained increasing appreciation in extramedullary haematopoiesis (EMH) and neutrophilia in tumors, which were later shown to possess abnormal myeloid-cell differentiation. Originally, these abnormal myeloid cells were described as myeloid progenitor cells, which could inhibit lymphocyte numbers and cytotoxic T lymphocyte (CTL) activity [22]. In 1987, hematopoiesis and suppressor bone marrow cells were first observed in patients with Lewis lung carcinoma [23]. In 1996, these cells became known as myeloid-derived suppressor cells. At present, it was clear that these cells lacked membrane markers which express on the surface of mature T-cells, B-cells, and natural killer (NK) cells, as well as macrophages [24]. For several decades, the understanding of MDSCs ranged from “the abnormal myeloid cell” to “immature myeloid cells” to “myeloid-derived suppressor cells.” From recent studies, we now know that MDSCs are a heterogeneous population of cells that have an immature state and a remarkable ability to suppress T-cell responses [12]. They are an intrinsic part of the myeloid-cell lineage comprising myeloid-cell progenitors and precursors of myeloid cells. The functional importance of MDSCs in the immune system has received attention over the last decade, and recent spotlights can be attributed to MDSCs's role in the negative regulation of immune responses during cancer, other chronic diseases, and sepsis [13].

Relevant in vivo and in vitro studies have shown that approximately 1–5% of MDSCs form myeloid-cell colonies, and about one-third of this population have the ability to differentiate into mature macrophages and DCs in the presence of appropriate cytokines [25, 26]. In mice, MDSCs are characterized by the coexpression of the myeloid lineage differentiation antigens, Gr-1 and CD11b [27]. Normal murine bone marrow contains 20–30% of cells with this phenotype, but only a small proportion (2–4%) is present in the spleen cells. In sepsis, approximately 40% of the splenocyte population, approximately 90% of cells in bone marrow, and 3–5% cells in peripheral lymph nodes coexpress Gr-1 and CD11b [13]. Conversely, MDSCs in humans do not express this phenotype, rather the cells are most commonly defined as CD14^−^CD11b^+^ cells. Human MDSCs may express the common myeloid marker CD15 or CD33 but lack the expression of markers of mature myeloid and lymphoid cells and the MHC-class-II molecule HLA-DR [28–30]. The cells described as MDSCs in human studies comprise only 0 to 0.5% of peripheral blood mononuclear cells.

Recently, many studies indicate that MDSCs can be delineated into two types: granulocytic MDSCs and monocytic MDSCs. Granulocytic-MDSCs have a CD11b^+^Ly6G^+^Ly6C^low^ phenotype, whereas monocytic MDSCs are CD11b^+^Ly6G^−^Ly6C^high^ [31]. These two different phenotypes possess different functions in the pathophysiology of sepsis. Firstly, only monocytic MDSCs have the ability to differentiate into mature DCs and macrophages in vitro. Furthermore, the granulocytic subset of MDSCs was found to express high levels of reactive oxygen species (ROS) and low levels of nitric oxide (NO), whereas the monocytic subset expressed the opposite pattern. Both subsets expressed arginase 1 (Arg-1) [31]. The abilities of the two cell types to suppress T-cell activity are also different. Monocytic MDSCs produced NO and strongly inhibited T-cell proliferation, while granulocytic-MDSCs produced low levels of NO and did not inhibit T-cell proliferation [32]. The specific difference between the two MDSCs subsets remains to be elucidated.

## 4. The Activation and Mechanisms of MDSCs

**Figure 1:**
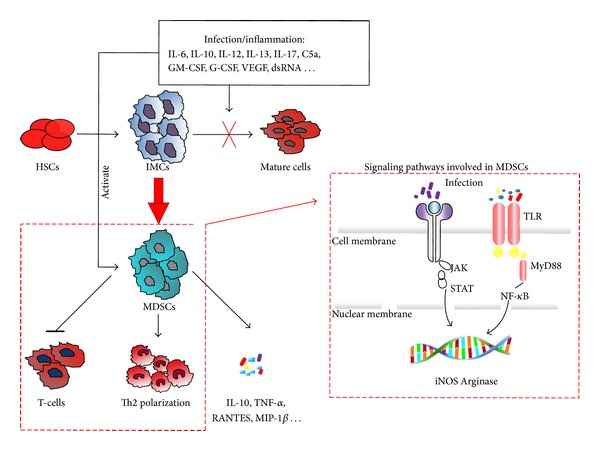
The origin and signaling pathways involved in MDSCs in sepsis. Haematopoietic stem cells (HSCs) differentiate into immature myeloid cells (IMCs) and then quickly differentiate into mature granulocytes, macrophages, or dendritic cells (DCs). In septic conditions, inflammatory factors such as IL-6, IL-10, IL-12, G-CSF, ds RNA, VEGF, and GM-CSF are elevated. They prevent IMCs from differentiating into mature myeloid cells. MDSCs expansion and activation is regulated by many signaling pathways, such as a toll-like receptor (TLR) mediated myeloid differentiation primary response gene 88 (MyD88) signaling and the granulocyte-colony stimulating factor receptor (G-CSFR) mediated the Janus kinase (JAK)/signal transducer and activator of transcription (STAT) pathway. They contribute to the increased production of reactive oxygen species (ROS), inducible nitric oxide synthase (iNOS), and arginase 1 (ARG1). MDSCs in sepsis can reduce the capacity of septic monocytes, macrophages, and neutrophils to respond to bacterial toxins, inhibit the activation of T-cells, and promote Th2 polarization. In addition, MDSCs can secrete several cytokines and chemokines, such as interleukin 10, TNF-*α*, RANTES, and MIP-1*β*.

In healthy individuals, immature myeloid cells (IMCs) are generated in bone marrow and quickly differentiate into mature granulocytes, macrophages, or dendritic cells (DCs). In septic conditions, inflammatory factors such as IL-6, IL-10, IL-12, G-CSF, dsRNA, IFN-*γ*, VEGF, and GM-CSF are elevated, which prevents IMCs from differentiating into mature myeloid cells [33] (Figure 1). In sepsis, MDSC expansion is regulated by many factors, and these factors trigger several different signaling pathways. GM-CSF and IFN-*γ* have the potential to induce toll-like receptor (TLR) mediated myeloid differentiation primary response gene 88 (MyD88) signaling. Granulocyte-colony stimulating factor (G-CSF) and its receptor initiate the Janus kinase (JAK)/signal transducer and activator of transcription (STAT) pathway [34–36] (Figure 1). These factors not only improve the accumulation of MDSCs, but also initiate their activation.

The most important function of MDSCs is to inhibit immune response via suppressing T-cell proliferation and activation [37]. It has been shown that MDSCs mediate their effect on T lymphocytes in cancer through direct contact and/or through a combination of multiple major mediators such as inducible nitric oxide synthase (iNOS), arginase-1 (Arg1), reactive oxygen species (ROS), transforming growth factor-*β* (TGF-*β*), IL-10, regulatory T-cells (Treg), and macrophages [38]. The following is a summary of the mechanisms of action of these mediators.

Arg1 and iNOS are expressed highly in monocytic-MDSCs and utilize L-arginine to produce urea and NO, respectively. Monocytic-MDSCs inhibit T-cell responses through the depletion of L-arginine via the two enzymes. The activation of either of these enzymes inhibits T-cell proliferation by interfering with the transduction of intracellular signals and by inducing T-cell apoptosis [39]. In vitro, iNOS inhibitors (L-NMMA) alone and in combination with Arg1 inhibitors block inhibition of T-cells by MDSCs. Similarly, phosphodiesterase-5 inhibitors delay tumor progression by decreasing Arg1 and iNOS expression and by regulating the suppressive machinery of MDSCs [40]. ROS production has been shown to be a major regulator of the suppressive activity of the granulocytic-MDSCs in both murine models and human cancers [41, 42]. In three different studies, inhibition of ROS production was associated with complete elimination of the suppressive activities of the MDSCs that were isolated from mice and human cancers [28, 41]. In addition, the combination of NO and ROS was associated with the production of peroxynitrite. Peroxynitrite causes protein dysfunctions in target cells and nitration of the T-cell receptor, which in turn, leads to suppression of CD8^+^ T-cell responses [43].

TGF-*β* is an immunosuppressive cytokine that has been firmly associated with MDSCs function and with the regulation of tumor induction and expansion [44]. In a study of squamous cell carcinoma of the head and neck, the CD14^+^HLA-DR^−^ MDSCs subset was noted to be the most predominant and produced higher levels of TGF-*β* compared with other MDSCs subsets [45]. TGF-*β* antibody partially restored T-cell proliferation and IFN-*γ* production. This evidence indicates that MDSCs are likely to be a major source for TGF-*β* production and their immunosuppressive effect is mediated by factors including TGF-*β* [45]. In a separate study, Lu et al. reported that TGF-*β* production promoted tumor cell invasion and metastasis [46]. Yang et al. found that the deletion of TGF-*β* receptor gene type II resulted in the infiltration of MDSCs in breast cancer and the production of large quantities of TGF-*β* that led to the promotion of tumor invasion and metastasis [47].

MDSCs may also inhibit T-cell proliferation indirectly by promoting the development of inducible CD4^+^CD25^+^Foxp3^+^ Treg. The development of Treg is linked to IL-10 plus TGF-*β* production [48]. Delano and coworkers have shown that MDSCs can still express several cytokines and chemokines, such as interleukin 10, TNF-*α*, RANTES, and MIP-1*β* [34]. High levels of CD80 expression by MDSCs were observed in many cancer tissues. Genetic knockout of CD80 expression in MDSCs alleviated the suppression of antigen-specific immune responses. CD80 itself suppressed antigen-specific immunity via Treg [49]. Another study analyzed the interaction of MDSCs with macrophages in a mouse cancer model and showed that, through IL-10 secretion, MDSCs also induced a type-2 polarization of macrophages which is characterized by a decrease of IL-12 secretion and that promotes tumor growth [50].

## 5. The Role of MDSCs in Sepsis

Although most of the current information about the function of MDSCs in immune responses has come from studies in the cancer field, there are increasingly more studies that directly investigate the roles of MDSCs in sepsis. Some researchers believe MDSCs are deleterious to the sepsis host. Delano et al. first demonstrated that MDSCs contribute to sepsis-induced T-cell suppression and preferential Th2 polarization [34]. They reported that GR-1^+^CD11b^+^ MDSCs population was dramatically increased in the spleen, lymph nodes, and bone marrow during polymicrobial sepsis. Phenotypically, these cells were heterogeneous, immature, and predominantly myeloid progenitors that express IL-10 and several other cytokines and chemokines. Splenic GR-1^+^ cells effectively decreased antigen-specific CD8^+^ T-cell IFN-*γ* production but only modestly suppressed antigen-specific and nonspecific CD4^+^ T-cell proliferation. GR-1^+^ cell depletion in vivo prevented both the sepsis-induced augmentation of Th2 cell-dependent and depression of Th1 cell-dependent antibody production. They further concluded that signaling through MyD88 is required for complete MDSCs expansion [34]. Martire-Greco and colleagues demonstrated that decreasing the number of viable MDSCs via all-trans retinoic acid improves immunocompetence in a murine model of lipopolysaccharide-induced immunosuppression [51].

Nevertheless, several reports demonstrated that the expansion of activated MDSCs during sepsis may actually be protective. Noel et al. found that when MDSCs were depleted by gemcitabine treatment, burned mice were highly susceptible to secondary pseudomonas aeruginosa infections [52]. Supporting these data, Delano and colleagues showed that lethality to pseudomonas pneumonia was increased early on after the induction of sepsis, but not later, coinciding with repopulation (activation) of Gr1^+^/CD11b^+^ cells [53]. Sander and colleagues found that adoptive transfer of MDSCs efficiently protected gp130-deficient mice from sepsis-associated mortality [54]. In addition, hepatic acute-phase proteins control innate immune responses during infection by promoting MDSCs function [54]. In order to be protective against sepsis, MDSCs require prolonged activation [55]. Derive et al. induced septic shock by caecal ligation and puncture in adult mice. They found that polymicrobial sepsis induced a progressive accumulation of MDSCs, mainly CD11b^+^Ly6G^+^Ly6C^−^ granulocytic-MDSCs, in spleens. MDSCs harvested at day 10 after the onset of infection were highly responsive to LPS in terms of cytokines secretion, NF-*κ*B activation, ROS production, and arginase 1 activity. MDSCs collected at day 3 responded poorly to the stimulus. By contrast, both day 3 and day 10 MDSCs were able to inhibit T-cell proliferation. Furthermore, adoptive transfer of day 10 MDSCs to septic mice attenuated peritoneal cytokine production, increased bacterial clearance, and dramatically improved survival rates [55]. In the same animal model, another research group had similar findings in terms of the duration needed for MDSCs to acquire their protective effect. Adoptive transfer of early (day 3) MDSCs from septic mice into naive mice after caecal ligation and puncture increased proinflammatory cytokine production and early mortality. Conversely, transfer of late (day 12) MDSCs from septic mice had the opposite effect. Early and late MDSCs studied ex vivo also differed in their inflammatory phenotypes. Early MDSCs expressed nitric oxide and proinflammatory cytokines, whereas late MDSCs expressed arginase activity and anti-inflammatory IL-10. They concluded that as the septic inflammatory process progresses, the heterogeneous MDSCs shift from being proinflammatory to anti-inflammatory [56]. Taken together, the role of MDSCs in sepsis is still controversial.

## 6. Therapy Targeting MDSCs in Sepsis

The treatment of sepsis is challenging and complex, thus, sepsis-related mortality remains high. The principles of the initial management approach are to provide cardiorespiratory resuscitation and to mitigate the immediate threats of uncontrolled infection. Many therapies could be applied to a patient with sepsis, including the use of intravenous fluids and vasopressors, oxygen therapy and mechanical ventilation, and organ function support and other intensive life support. New research has shown that most patients admitted to intensive care units for treatment of sepsis had unresolved septic foci postmortem, suggesting that patients were unable to eradicate invading etiologic pathogens and were highly susceptible to nosocomial organisms, or both [11]. Several clinical trials of drugs that boosted immunity suggested that therapies that improve host immunity might increase survival because immunosuppression has a central role in sepsis-related deaths. Sepsis can be thought of as a battle between the invading microbes and the host's immune response, with each side seeking success. In addition to studying current management approaches, immunotherapies for sepsis must also be examined, especially those focused on the regulation of MDSCs.

Many efforts have been made to target MDSCs in cancer. Both sepsis and cancer share many immunological defects, therefore, some investigators postulate that the recent success of several immunomodulatory drugs in cancer may provide potential immunostimulatory therapies for sepsis [57]. To mitigate the immunosuppressive activities of MDSCs, one of the effective strategies is to differentiate MDSCs into mature cells. All-trans retinoic acid (ATRA) at therapeutic levels has been shown to reduce MDSCs and induce MDSCs differentiation into dendritic cells and macrophages in cancer patients and mice [58]. Nefedova et al. suggested that an upregulation of glutathione synthesis and a reduction in ROS levels were the main mechanisms involved in ATRA-mediated MDSCs differentiation [59]. In a recent randomized clinical trial involving patients with small cell lung cancer (*n* = 41), systemic depletion of MDSCs using ATRA in combination with cancer vaccination led to a statistically significant improvement in the immune response to p53 vaccination in comparison with the vaccination-only group (20%, *P* = 0.02) [60]. In another clinical study, Mirza et al. reported that ATRA administration in patients with metastatic renal cell carcinoma markedly reduced the number of MDSCs (Lin^−^HLA-DR^−^CD33^+^). ATRA also improved the myeloid/dendritic cell ratio and the ability of patients' mononuclear cells to stimulate allogeneic T-cells, increased the dendritic cells/MDSCs ratio in the peripheral blood, and improved the T-cell immune response [61]. In addition, Martire-Greco and colleagues demonstrate that ATRA improves immunocompetence in a murine model of lipopolysaccharide-induced immunosuppression by decreasing the number of viable MDSCs [51].

Inhibition of the signal pathways that regulate the production of the suppressive factors of MDSCs is another promising approach. Sildenafil, a phosphodiesterase-5 inhibitor, reduced arginase 1 and nitric oxide synthase-2 expression in a mouse tumor model. It enhanced intratumoral T-cell infiltration and activation, reduced tumor outgrowth, and improved the antitumor efficacy of adoptive T-cell therapy. Furthermore, sildenafil restored T-cell proliferation of peripheral blood mononuclear cells from multiple myeloma and head and neck cancer patients in vitro [62]. It is not clear whether this favorable effect will be observed clinically in cancer patients. Nitroaspirin is a classic aspirin molecule covalently linked to an NO donor group and is able to release NO. Nitroaspirin does not possess direct antitumor activity. However, by interfering with the inhibitory enzymatic activities of MDSCs, orally administered nitroaspirin normalized the immune status of tumor-bearing hosts, increased the number and function of tumor-antigen-specific T lymphocytes, and enhanced the preventive and therapeutic effectiveness of the antitumor immunity elicited by cancer vaccination [63]. Recently, cimetidine, a histamine type-2 receptor antagonist, was shown to reduce NO production and arginase I expression of MDSCs. MDSCs were prone to apoptosis due to cimetidine treatment resulting in a reversal of MDSCs-mediated T-cell suppression and improved IFN-*γ* production [64].

Several studies in mice tested a “cell-based therapy” approach using MDSCs or MDSCs-like cells in the treatment of diabetes [65], immunological hepatic injury (IMH) [66], and graft-versus-host disease (GVHD) [39]. Yin et al. found that administration of MDSCs can prolong the survival of diabetic mice transplanted with allogeneic pancreatic cells [65]. Highfill et al. reported that the adoptive transfer of MDSCs significantly improved survival in a model of graft-versus-host disease [67]. In both of these studies, MDSCs were generated ex vivo by culturing BM cells with a combination of colony stimulating factors and interleukins.

Presently, there is no clinical trial targeting MDSCs in sepsis. Some experts believe that the absence of activated MDSCs in patients with sepsis might be the reason why these patients succumb to nosocomial infection [68]. Therefore, pharmacologic agents known to regulate the production of the suppressive factors of MDSCs or promote the expansion of MDSCs such as growth factors, chemokines, and sildenafil require further study in an effort to improve patient outcomes in sepsis.

## 7. Conclusions

In recent years, it has become clear that most septic patients do not die from an overwhelming proinflammatory immune response, but rather succumb to their illness in an immunosuppressive state. Although control of the infection and supportive therapies will remain the mainstay for treatment in the early phase of sepsis, there is a developing trend towards immunostimulation for patients in immunosuppressive states. MDSCs are a heterogeneous population of cells that have an immature state and the ability to suppress T-cell responses. The roles of MDSCs in sepsis remain uncertain. Some believe MDSCs are beneficial to the septic host and that the absence of activated MDSCs is the reason why some patients subsequently succumb to nosocomial infection. Others believe that MDSCs are deleterious and the expansion of MDSCs in the host following sepsis leads to global adaptive immune suppression and secondary infection. The roles and mechanisms of MDSCs warrant further exploration and MDSCs could serve as a viable target for sepsis treatment.
